# Pre-pyloric enteral nutrition versus total parenteral nutrition on survival in dogs with acute pancreatitis

**DOI:** 10.18849/ve.v10i4.730

**Published:** 2025-12-11

**Authors:** Felix Tsz Fung Ting+

**Keywords:** ACUTE PANCREATITIS, CANINE, DOG, MORTALITY, NUTRITION

## Abstract

**PICO question:**

In dogs with acute pancreatitis, does pre-pyloric enteral nutrition compared to total parenteral nutrition result in an improved survival rate?

**Clinical bottom line:**

**Category of research:**

Treatment.

**Number and type of study designs reviewed:**

One paper was critically reviewed. It was an open-label, prospective pilot study.

**Strength of evidence:**

Weak.

**Outcomes reported:**

There was no difference in outcome between the two treatments in this study.

**Conclusion:**

There is currently weak evidence on the effect of pre-pyloric enteral nutrition compared to total parenteral nutrition on the survival rate of dogs with acute pancreatitis.

## How to apply this evidence in practice

The application of evidence into practice should take into account multiple factors, not limited to: individual clinical expertise, patient’s circumstances and owners’ values, country, location or clinic where you work, the individual case in front of you, the availability of therapies and resources.

Knowledge Summaries are a resource to help reinforce or inform decision making. They do not override the responsibility or judgement of the practitioner to do what is best for the animal in their care.

## Clinical scenario

You diagnosed acute pancreatitis (AP) in a five-year-old Miniature Schnauzer with a three-day history of lethargy, inappetence, and vomiting. The dog was admitted to the hospital and supportive therapy was implemented. You are aware of the importance of nutritional support in critical patients; therefore, you are considering nutritional supplementation. You are uncertain whether to choose pre-pyloric enteral nutrition (PPEN) or total parenteral nutrition (TPN) to optimise the outcome. To make an informed decision, you begin researching evidence comparing the effects of the two treatment options on the survival rates in dogs with AP.

## The evidence

A literature review identified a single study meeting the criteria for review (Mansfield et al., 2011); this study did not find a statistically significant difference between survival rates between dogs administered PPEN and TPN (P = 1.0). Nonetheless, due to the small sample size (ten), this study has a high probability of type II error.

## Summary of the evidence

**Mansfield et al. (2011):** A Pilot Study to Assess Tolerability of Early Enteral Nutrition via Esophagostomy Tube Feeding in Dogs with Severe Acute Pancreatitis **Aim: **To evaluate the tolerability of administering pre-pyloric enteral nutrition compared with parenteral nutrition in dogs with severe acute pancreatitis.

Population:	Adult dogs that were admitted to Murdoch University Veterinary Hospital (Australia) for the treatment of acute pancreatitis (AP). Dogs were excluded if they did not meet the inclusion criteria.
Sample size:	10 dogs.
Intervention details:	All dogs received intravenous fluid therapy, antiemetics, analgesia treatment, a bolus of electrolyte solution via a nasooesophageal tube, and fresh frozen plasma. Broad-spectrum antibiotics were given if dogs had a neutrophil count of less than 2.0 x 10^9/L or a left shift combined with pyrexia (over 39 °C). Dogs were consecutively assigned to one of the two treatment groups and anaesthetised for placement of either an oesophagostomy tube receive pre-pyloric enteral nutrition (PPEN), or a jugular catheter placement to receive total parenteral nutrition (TPN). Dogs receiving PPEN (n = 5) were fed ⅓ of their daily calculated caloric requirement (resting energy requirement (RER) x 1.25) on day 1, ⅔ on day 2 and 100% on day 3. The diet consisted of low-fat commercial dog food, enteric-coated pancreatic enzymes and medium-chain triglyceride oil. Dogs receiving TPN (n = 5) were given 50% RER on day 1 and 100% on day 2 until day 3. 17.5% of calories were supplied as lipid, 70.2% as 50% dextrose and 12.3% as amino acid solution. After day three, the nutritional intervention of all dogs was based on individual requirements instead of their assigned groups.
Study design:	Open-label, prospective pilot study.
Outcome Studied:	The number of vomiting or regurgitation episodes in each treatment group (analysed by a Poisson model), which is irrelevant to the PICO question. The proportion of dogs that survived after each treatment was compared via Fisher’s exact test with significance set at P < 0.05 by a 2-sided probability.
Main Findings (relevant to PICO question):	All dogs that received PPEN survived (5/5) and four dogs that received TPN survived (4/5). This was not statistically significant (P = 1).
Limitations:	The small sample size limited the strength of the result; a larger size of at least 86 dogs is required to generate a statistically significant conclusion for the PICO question. The lack of randomisation might introduce selection bias, negatively affecting the study's validity.

## Appraisal, application and reflection

Canine acute pancreatitis (AP) has a moderate mortality rate, estimated at 27% to 58%, with sample sizes ranging from 30 to 101 dogs (Jensen & Chan, 2014; Mansfield, 2012; Mansfield & Beths, 2015). Although idiopathic AP is the most common cause, several risk factors have been identified, including dietary components, drugs or toxins (e.g. phenobarbitone, L-asparaginase), endocrinopathies, hypertriglyceridaemia, and genetics (Lim et al., 2024). In addition to supportive therapies such as intravenous fluids, antiemetics, gastric protectants, and antibiotics (when indicated) (Lim et al., 2024; Mansfield & Beths, 2015), nutritional management also plays an important role in the management of AP (Jensen & Chan, 2014). Dogs with AP have increased nutrition requirements since AP is a catabolic disease leading to significant nitrogen loss. Moreover, AP often results in ileus, which reduces voluntary diet intake (Sax et al., 1987; Thomson, 2006).

The two primary nutritional approaches in anorexic AP cases are enteral nutrition (EN), provided through the gastrointestinal tract, and total parenteral nutrition (TPN), delivered intravenously via a central line as a complete nutritional substitute (Chan et al., 2002). Enteral nurtition can be further classified into pre-pyloric EN (PPEN), administered via nasogastric, oesophagostomy, or gastrostomy tubes, and post-pyloric EN, delivered through intrajejunal or nasojejunal tubes. Although it is believed that post-pyloric EN avoids the stimulation of pancreatic secretions and is less susceptible to the effects of nausea or vomiting (Heuter, 2004; Hinden et al., 2020), the treatment is not widely accessible in general practice and referral hospitals in the UK since it requires more technical expertise. Additionally, the benefits of post-pyloric EN over PPEN in AP management lack evidence in canine patients, and studies in human AP show no significant differences in discharge rates, surgical needs, or mortality between post-pyloric EN and PPEN (Chang et al., 2013; Eatock et al., 2005; Kumar et al., 2006).

This PICO question asks if dogs with AP experience improved survival rates with PPEN compared to. Traditionally, TPN was favoured over PPEN in AP management in dogs due to concerns that PPEN might stimulate pancreatic enzyme secretion and exacerbate auto-digestion (Simpson & Lamb, 1995; Stabile et al., 1984; Williams, 1994). In contrast to this belief, research in humans, laboratory rats, and dogs has shown that pancreatic secretion is reduced during AP, and damaged acinar cells have a diminished response to physiological stimuli (Boreham & Ammori, 2003; Niederau et al., 1990; O’Keefe et al., 2005; Qin et al., 2003). Moreover, the gastrointestinal tract is now recognised as a major contributor to the systemic inflammatory state in veterinary patients with AP, particularly when luminal nutrients are lacking (Flint & Windsor, 2003). Total parenteral nutrition, on the other hand, can cause complications such as hyperglycaemia and lipaemia, which can further complicate the management of AP in dogs (Chan et al., 2002; Freeman et al., 1995; Reuter et al., 1998).

During the literature review, several original research articles were excluded. One retrospective study found that compared to TPN, dogs receiving both parenteral nutrition and PPEN had a statistically higher survival rate (Chan et al., 2002). However, this study was rejected as it did not distinguish data relating to AP from other diseases. Two experimental studies compared intrajejunal EN and TPN in surgically induced AP in laboratory dogs (Qin et al., 2002; Xu et al., 2006) found reduced endotoxin and gut bacterial translocation in dogs receiving EN compared to TPN. The authors also suggested that EN facilitates the maintenance of intestinal mucosal barrier integrity, as they observed an increase in mucosal protein and DNA contents, height of villi and thickness of the mucosa and intestinal wall in dogs receiving EN (Qin et al., 2002; Xu et al., 2006). While these findings suggest that EN provides benefits over TPN and could potentially improve survival rates in dogs with AP, both studies were excluded because they employed intrajejunal EN instead of PPEN and did not compare the outcomes of the dogs in the experiment. Only one study met the inclusion criteria (Mansfield et al., 2011). While the findings indicated a significantly lower incidence of vomiting or regurgitation in dogs with AP receiving PPEN compared to TPN (P < 0.001), no difference in overall outcomes was observed (P = 1) (Mansfield et al., 2011). Although the study suggested no significant benefit in survival rates when TPN was administered instead of PPEN, its statistical power was limited by the small sample size. Based on the binomial survival proportions of 5/5 in the PPEN group versus 4/5 in the TPN group, Mansfield et al. (2011) calculated that a sample size of 43 dogs per group would be required to detect a statistically significant difference with 95% confidence and 80% power. If the TPN to PPEN ratio is 2:1, the required sample size would be 35 dogs in the PPEN group and 70 in the TPN group (Mansfield et al., 2011).

The PICO question broadly categorises nutritional supplementation into PPEN and TPN, allowing a straightforward comparison of survival rates in dogs with AP between the two methods. However, it overlooks the variability within each method. For instance, the dietary composition of PPEN varies from elemental diets to complete liquid foods (Jensen & Chan, 2014; Mansfield, 2012; Simpson & Lamb, 1995; Stabile et al., 1984; Thomson, 2006). Although TPN primarily supplies macronutrients such as carbohydrates, proteins, and lipids, studies suggest varying macronutrient compositions (Chan et al., 2002; Qin et al., 2003; Reuter et al., 1998; Sax et al., 1987; Stabile et al., 1984; Thomson, 2006). To the knowledge of the author of this Knowledge Summary, there is weak evidence to identify the optimal constitution for PPEN or TPN. Given this variability, follow-up PICO questions, such as ones that compare the outcomes of different TPN or PPEN nutritional compositions, would provide valuable insights for veterinary practitioners in selecting the most appropriate dietary strategy for dogs with AP.

## Methodology

**Search Strategy:** 

Databases searched and dates covered:	CABI Digital Library on Atypon Literatum (1973 to 04 Mar 2025) PubMed on NCBI website (1973 to 04 Mar 2025)
Search strategy:	CABI Digital Library: pancreatitis AND (dog OR cani* OR pupp* OR pup OR bitch) AND (parenteral) AND (enteral OR pre-pyloric OR prepyloric OR nasoesophageal OR nasooesophageal OR nasogastric OR oesophagostomy OR esophagostomy OR gastrostomy) PubMed: pancreatitis AND (dog OR cani* OR pupp* OR pup OR bitch) AND (parenteral) AND (enteral OR pre-pyloric OR prepyloric OR nasoesophageal OR nasooesophageal OR nasogastric OR oesophagostomy OR esophagostomy OR gastrostomy)
Dates searches performed:	04 Mar 2025

**Exclusion / Inclusion Criteria:** 

Exclusion:	Research studies irrelevant to the PICO question. Non-canine literature. Studies unavailable in English.
Inclusion:	Peer-reviewed studies.

**Search Outcome:** 

Database	Number of results	Excluded – not in English	Excluded – human literature	Excluded – research studies that were irrelevant to the PICO question	Total relevant papers
CABI Digital Library	21	0	7	13	1
PubMed	20	1	6	12	1
Total relevant papers when duplicates removed					1

## Acknowledgements

I would like to thank Catriona Pennington for supervising the writing of this article.

## ORCiD

Felix Tsz Fung Ting: https://orcid.org/0009-0007-6238-0896

## Conflict of Interest

The author declares no conflicts of interest.
